# Ghrelin Drives Food Intake by Attentional Bias Toward Food Cues

**DOI:** 10.1002/cph4.70281

**Published:** 2026-10-04

**Authors:** Renée Poelman, Marie V. Le May, Iris Stoltenborg, Edoardo Vecchi, Erik Schéle, Roger A. H. Adan, Suzanne L. Dickson

**Affiliations:** ^1^ Department of Physiology/Endocrine, Institute of Neuroscience and Physiology The Sahlgrenska Academy at the University of Gothenburg Gothenburg Sweden; ^2^ Department of Translational Neurosciences University Medical Center Utrecht Utrecht the Netherlands

**Keywords:** attention, FED3, food cues, ghrelin

## Abstract

Food‐predictive cues can promote eating, but the contribution of metabolic signals to cue‐guided behavior remains incompletely understood. Ghrelin is an orexigenic hunger hormone that has previously been linked to behavioral and neural responses to food‐predictive cues, although many paradigms cannot distinguish general hunger‐driven intake from intake expressed in response to discrete conditioned cues. We developed a home‐cage operant task using the Feeding Experimentation Device 3 (FED3) to assess attention‐linked responding to an illuminated food‐predictive food cue in male mice. Following staged training, eight mice were tested under fed, overnight‐fasted and ghrelin‐treated conditions in a crossover design. Fasting and ghrelin increased correct and premature responses, and reduced omissions, indicating enhanced engagement with the conditioned food cue, with motivational and impulsivity‐related processes likely contributing to the behavioral profile. Ghrelin increased food intake during the cue‐guided task but not during FreeFeeding in the absence of the illuminated predictive cue, whereas fasting increased intake in both contexts. The maximum obtainable food intake was elevated by fasting and ghrelin, but only during the cue‐guided task. These findings establish a home‐cage FED3‐based paradigm for resolving multiple attention‐linked components of food‐cue responding and show that acute ghrelin and fasting produce overlapping effects on cue‐guided engagement but differ in their effects on food intake across task contexts, with ghrelin preferentially increasing intake when food availability is signaled by a conditioned cue.

## Graphical abstract

Fasting and ghrelin enhanced engagement with food‐predictive cues in a home‐cage FED3 task, increasing correct and premature responses while reducing omissions. However, ghrelin preferentially increased food intake when food availability was signaled by a predictive cue, whereas fasting increased intake regardless of cue context.

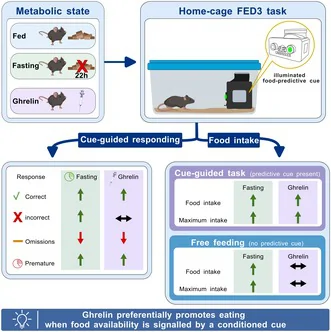

## 1 Introduction

Overweight and obesity are major risk factors for cardiovascular disease and represent a growing global health challenge. Among environmental drivers, food‐predictive cues such as sight, smell or advertisements, play a key role in promoting overeating and weight gain (Boswell and Kober 2016; Swinburn et al. 2011). In rodent studies, conditioned cues predicting food availability accelerate feeding (Weingarten 1983) and can drive intake even in sated animals (Petrovich and Gallagher 2007; Weingarten 1983). Similar effects have been reported in humans, where exposure to palatable foods increases the desire for and intake of those foods (Cornell et al. 1989) and exposure to food advertisements increased food intake in both children and adults (Harris et al. 2009).

Food‐cue reactivity encompasses the behavioral, physiological and neural responses elicited by food‐related cues. These responses include activation of reward‐ and sensory‐related brain circuits, endocrine and autonomic responses, and behavioral actions that ultimately contribute to food seeking and consumption (Boswell and Kober 2016). Human neuroimaging studies consistently show that exposure to food cues activates brain areas involved in visual processing, taste, reward, impulsivity and attention (van der Laan et al. 2011; Yokum et al. 2011). There are indications that attentional focus may play a greater role in the neural response to visual food cues than palatability of the food itself (Franssen et al. 2020; Pimpini et al. 2022). Within this broader framework, attentional processes are thought to play an important role by influencing the extent to which food cues capture and maintain behavioral engagement. Behavioral studies demonstrate attentional biases toward food‐related stimuli, that are influenced by factors such as food palatability and energy content of the food depicted in the images (di Pellegrino et al. 2011; Doolan et al. 2014; van der Laan et al. 2011) as well as individual differences in cue‐responsiveness and impulsivity (Brignell et al. 2009; Hou et al. 2011). Thus, attentional allocation represents one component through which food‐predictive cues may influence feeding behavior.

Food‐cue responsiveness is not fixed but varies according to internal physiological state. In humans, attentional bias toward food‐related stimuli differs between hunger and satiety and is influenced by both body weight status and metabolic condition (Castellanos et al. 2009; Nijs et al. 2010; Schlezingerová et al. 2024). Neuroimaging studies similarly indicate that responses to visual food cues are sensitive to fasting state, with hunger enhancing activation of brain regions involved in reward, motivation and food‐cue processing (Kroemer et al. 2013; Wever et al. 2021). Thus, signals generated during fasting and negative energy balance interact with food‐predictive cues to shape behavioral responding.

Among the physiological signals that change during negative energy balance, the stomach‐derived hormone ghrelin (Kojima et al. 1999) has emerged as a plausible modulator of food‐cue responsiveness. Circulating ghrelin levels rise during fasting in both humans and rodents (Muller et al. 2002; Tschöp et al. 2000), rise before meals (Cummings et al. 2001) and are closely associated with subjective hunger (Cummings et al. 2004). Beyond its well‐established orexigenic effects in humans and rodents (Wren, Seal, et al. 2001; Wren, Small, et al. 2001; Wren et al. 2000) ghrelin signaling contributes to anticipatory and reward‐related aspects of feeding (Egecioglu et al. 2010; Merkestein et al. 2012; Perello and Dickson 2015; Perello et al. 2010; Skibicka and Dickson 2011; Skibicka et al. 2011; Skibicka et al. 2012). Kanoski and colleagues showed that ventral hippocampal ghrelin signaling promotes learned and motivational aspects of feeding elicited by a conditioned stimulus previously paired with meal access (Kanoski et al. 2013). Also, disruption of ghrelin signaling reduces cue‐potentiated feeding, although the pattern of disruption differs between mice lacking GHSR and mice receiving pharmacological GHSR antagonism (Walker et al. 2012). Ghrelin has also been shown to regulate phasic dopamine and nucleus accumbens signaling evoked by food‐predictive stimuli (Cone et al. 2015), further supporting a role for ghrelin in cue‐related neural processing. Furthermore, exposure to food cues increases circulating ghrelin levels in both rodents and humans (Peris‐Sampedro et al. 2021; Schüssler et al. 2012) and higher ghrelin concentrations are associated with enhanced neural responses to food cues in humans (Kroemer et al. 2013; Malik et al. 2008; Wever et al. 2021). Together, these findings identify ghrelin as a candidate signal through which metabolic state may influence behavioral responsiveness to food‐predictive cues.

Although these findings implicate ghrelin in food cue‐responsiveness, how this translates into behavior remains incompletely understood. In particular, paradigms used to demonstrate ghrelin‐induced feeding involve food that is visible, accessible or embedded within a food‐associated context (Bake et al. 2020; Egecioglu et al. 2010; Peris‐Sampedro et al. 2021; Schéle et al. 2016; Skibicka et al. 2011, 2012; Wren, Small, et al. 2001), making it difficult to distinguish generalized hunger‐driven intake from behavior directed toward discrete conditioned cues. In the present study, we developed a home‐cage FED3‐based operant task to assess attention‐linked behavioral responses to a conditioned food cue in mice. Using this approach, we compared the effects of fasting and acute ghrelin administration on multiple components of cue‐guided performance, including correct responses, omissions, premature responses and food intake. We additionally compared intake during the cue‐guided task with intake during a “FreeFeeding” task, where food is freely available in the absence of the illuminated predictive cue, allowing us to examine whether fasting‐ and ghrelin‐induced feeding were expressed similarly across task contexts.

## 2 Materials & Methods

### 2.1 Animals

Adult male C57BL/6J mice (Charles River Laboratories, Sulzfeld, Germany) were used (aged 8 weeks at the start of the experiment). Mice were group housed (8 mice per cage) up to 1 week before the first experimental procedure, after which they were single housed and acclimated for 1 week. Housing conditions were 20°C–22°C, 50% humidity under a reversed 12‐h dark–light cycle (lights on at 04:00 p.m.). Unless stated otherwise, mice had ad libitum access to water and standard chow (2016 Teklad diet; Envigo, Cambridgeshire, UK; 3.0 kcal/g). All procedures were approved by the local Ethics Committee for Animal Care in Gothenburg, Sweden (Göteborgs djurförsöksetiska nämnd; permit number 2021‐3400, approved 6 December 2021) and conducted in accordance with the EU Directive 2010/63/EU.

### 2.2 Behavioral Procedures

#### 2.2.1 Apparatus

Behavior was assessed using the Feeding Experimentation Device 3 (FED3; Open Ephys Production Site, Lisbon, Portugal) which measures food intake and operant responses in the home‐cage. This provides multiple advantages over classic operant responding boxes since it allows for longer training sessions and measurements with minimal experimenter intervention (Matikainen‐Ankney et al. 2021). It also allowed us to compare intake during an illuminated cue‐guided operant task with intake during FreeFeeding lacking the illuminated predictive cue. FED3 features two nose‐poke ports with neopixel LED lights (multi‐color, set to green, brightness 40) and a dispenser that delivers 20 mg chow pellets into a magazine located in between the two ports (Figure 1B). All events, including nose pokes and pellet retrieval, were logged to an internal microSD card. Using the FED3 Arduino library (v1.16.2) and Arduino IDE (v1.8.16), we implemented a custom cue‐guided operant program in which a nose poke at the illuminated port triggered pellet delivery. For continuous food intake measurements, we utilized the “FreeFeeding” FED3 example program, which dispenses a new pellet after the previous pellet is removed from the magazine.

**FIGURE 1:**
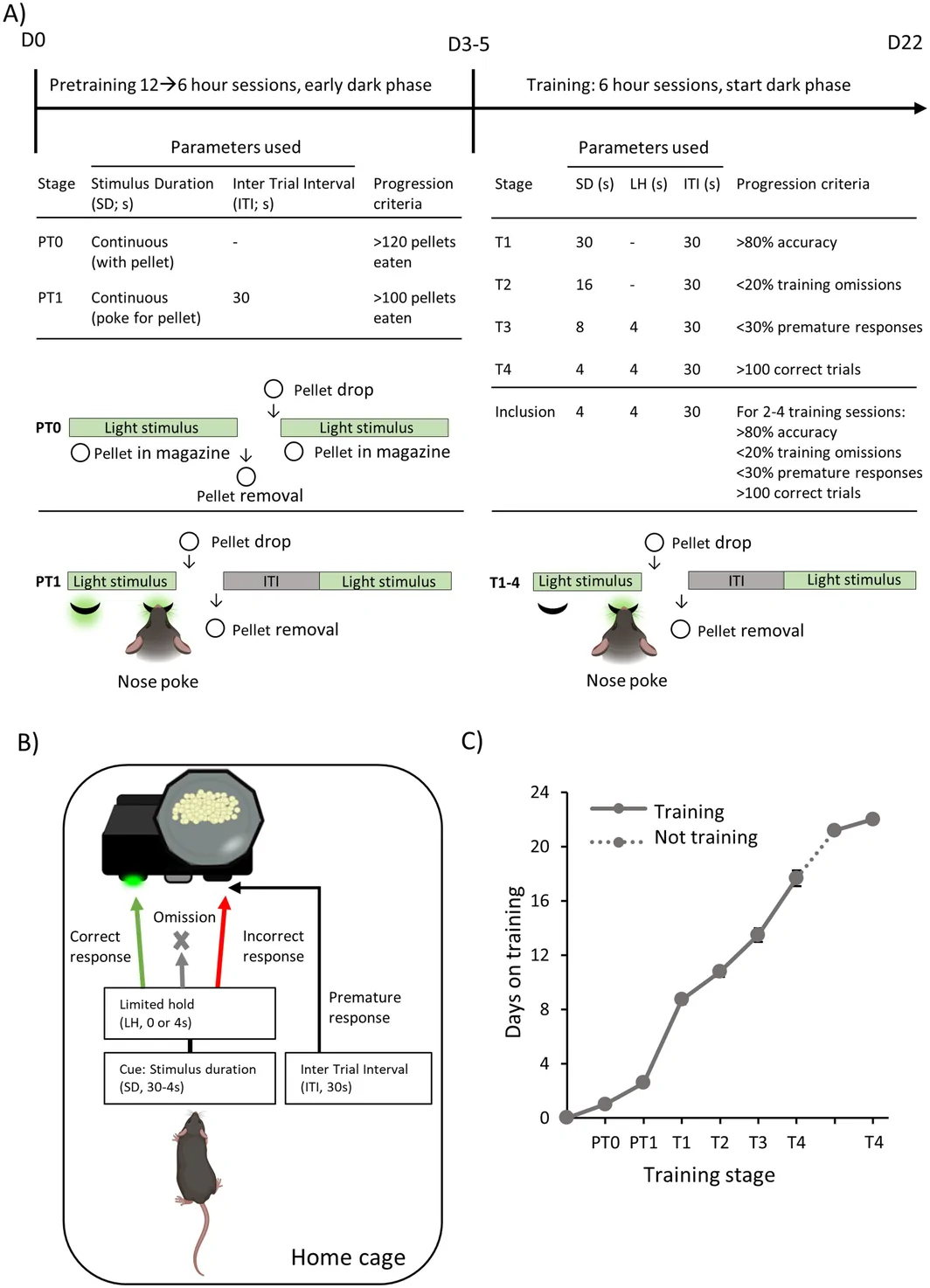
Establishment of the attention paradigm in mice. (A) Training schedule and schematic overview of task stages, including stimulus parameters (stimulus duration, SD; inter‐trial interval, ITI; limited hold, LH), progression‐ and inclusion criteria. During PreTraining0 (PT0), continuous illumination of nose poke holes signaled pellet availability; pellet removal initiated a brief (1–2 s) lights‐off ITI while dispensing a new pellet. In PreTraining1 (PT1), a nose poke into either illuminated hole triggered pellet delivery, followed by a 30 s ITI during which responses were not reinforced. In Training stages 1–4 (T1–4), one nose poke hole was illuminated for a limited SD, alternating sides. Responses to the illuminated hole were reinforced, followed by a 30 s ITI. (B) Schematic representation of response types: Correct, incorrect, omission, and premature responses. Only a correct response results in pellet delivery. (C) Average number of daily sessions required to complete each training stage (*n* = 8; mean ± SEM).

#### 2.2.2 Training Conditions

15 mice entered training. Training occurred during the dark phase when mice naturally consume most of their daily food intake (Possidente and Birnbaum 1979). Sessions lasted for 6 h, except for initial habituation (pretraining 0, PT0) which lasted 12 h (Figure 1A). Outside training sessions, FED3 devices remained inactive and were removed only briefly for data retrieval or charging. During training, mice could complete unlimited trials and obtained all food from the FED3. Food intake and body weight were monitored daily. Mice completing < 100 trials (equivalent to 2 g chow) or falling below 90% baseline bodyweight received supplemented chow (~1.5 g), although this was rarely required. Pellet hoarding was not observed during daily cage checks.

Pretraining (PT) comprised two adaptation stages to establish magazine feeding and nose poking for food pellets, while also associating food availability with the illuminated ports (Figure 1A). In PT0, pellets were continuously dispensed into the magazine after removal of the previous pellet and both ports were illuminated to signal pellet availability. In PT1, both ports were illuminated and a nose poke into either illuminated port triggered pellet delivery and simultaneously extinguished the light. Pellet removal initiated a 30 s timeout or “Inter Trial Interval” (ITI) followed by the start of a new trial. Thus, mice learned to nose poke for pellet delivery while associating the illuminated ports with food availability. Responses in non‐illuminated ports during the ITI were counted but had no further programmed consequences. Criteria for progression to the next training phase are shown in Figure 1A. Mice typically completed pretraining in 1–3 days (Figure 1C).

#### 2.2.3 Attention Protocol

Unlike conventional operant tasks that require trial initiation, our protocol presented cues passively, mimicking real‐life exposure to environmental food‐predictive cues. In the first training phase (TR1), each trial began with a 30 s stimulus (stimulus duration, SD) in one of the two nose poke ports, alternating pseudorandomly between sides (Figure 1A). A correct response, defined as a poke in the illuminated port, triggered pellet delivery, extinguished the cue light and, with pellet retrieval, initiated a 30 s ITI (Figure 1B). Incorrect responses, defined as pokes in the non‐illuminated port, and premature responses, defined as pokes during ITI, were logged but had no further programmed consequences (Figure 1B). During training stages, omissions, defined as no response during the SD, were recorded only if the mouse was engaged in the task, operationalized as < 2‐min inactivity. Progression criteria for the next training stage were: > 80% accuracy (#correct response/(#correct + incorrect response) × 100%), < 20% training omissions (#training omissions/#trials × 100%), < 30% premature responses (#premature pokes/#total pokes × 100%) and > 100 correct trials.

Subsequent training phases (TR2–TR4) progressively reduced SD to 16, 8 and 4 s (Figure 1A). To ensure mice had sufficient time to respond to the shorter SD, nose pokes during a 4 s limited hold (LH) following cue extinction in TR3 and TR4 were registered as correct responses. Mice achieving stable TR4 performance for 2–4 days were included in the experiments. To avoid over‐training, qualified mice were temporarily returned to regular chow for 3–4 days, followed by two additional TR4 sessions before testing, during which they again met inclusion criteria (Figure 1C).

#### 2.2.4 Attention Challenge

The attention challenge task mirrored the final training stage but used variable stimulus durations (SD = 0.5–2 s) and inter‐trial intervals (ITI = 30–60 s) to increase attentional and impulse‐control demands. Trials were pseudorandomized for stimulus duration, ITI and side to ensure task unpredictability. Behavioral metrics included accuracy, omissions, premature responses, and pellet retrieval latency (Figure 1B). Mice were exposed to the attention challenge task for one 6 h habituation session before experimental testing.

### 2.3 The Effect of Fasting or Ghrelin on Attention‐Linked Responding to a Conditioned Food Cue

Following completion of the final training stage, mice received ad libitum access to regular chow and FED3 devices were removed from the home‐cage. To assess whether ghrelin or fasting affected attention‐linked responding to a conditioned food cue, mice were tested in three conditions: fed, fasted and ghrelin‐treated. In the fasted condition mice were food deprived for 22 h before testing. In the Fed and ghrelin‐treated conditions regular chow was removed 2 h prior to testing. Mice were exposed to the attention challenge task for 30 min. At 15 min before testing (ZT16), fasted mice received a subcutaneous (s.c.) saline injection, while fed mice received either s.c. saline or ghrelin (3 mg/kg dose; #1465; Tocris, Bristol, UK). In crossover experiments, mice were allowed two washout days between treatments and to recover from fasting.

### 2.4 The Effect of Fasting or Ghrelin on Intake During Cue‐Guided Testing and FreeFeeding

To assess whether the effects of fasting or ghrelin on attention‐linked behaviors were accompanied by changes in food intake, mice completed both cue‐guided sessions (attention task) and FreeFeeding sessions using the FED3 ‘FreeFeeding’ program. In the cue‐guided sessions, pellet delivery depended on a correct response to the illuminated predictive cue. In the FreeFeeding sessions, pellets were delivered without presentation of the illuminated predictive cue. We therefore refer to this condition as FreeFeeding lacking the illuminated predictive cue rather than as completely cue‐free feeding, since dispensed pellets may still provide olfactory, visual or tactile cues after delivery. Food intake was measured following fasting or ghrelin administration in both task contexts. The order of task exposure was counterbalanced and mice switched only once between paradigms to avoid confusion (Figure 2A). Mice exposed to FreeFeeding sessions before the cue‐guided sessions were re‐habituated to the attention task for one additional session prior to the cue‐guided experimental days.

**FIGURE 2:**
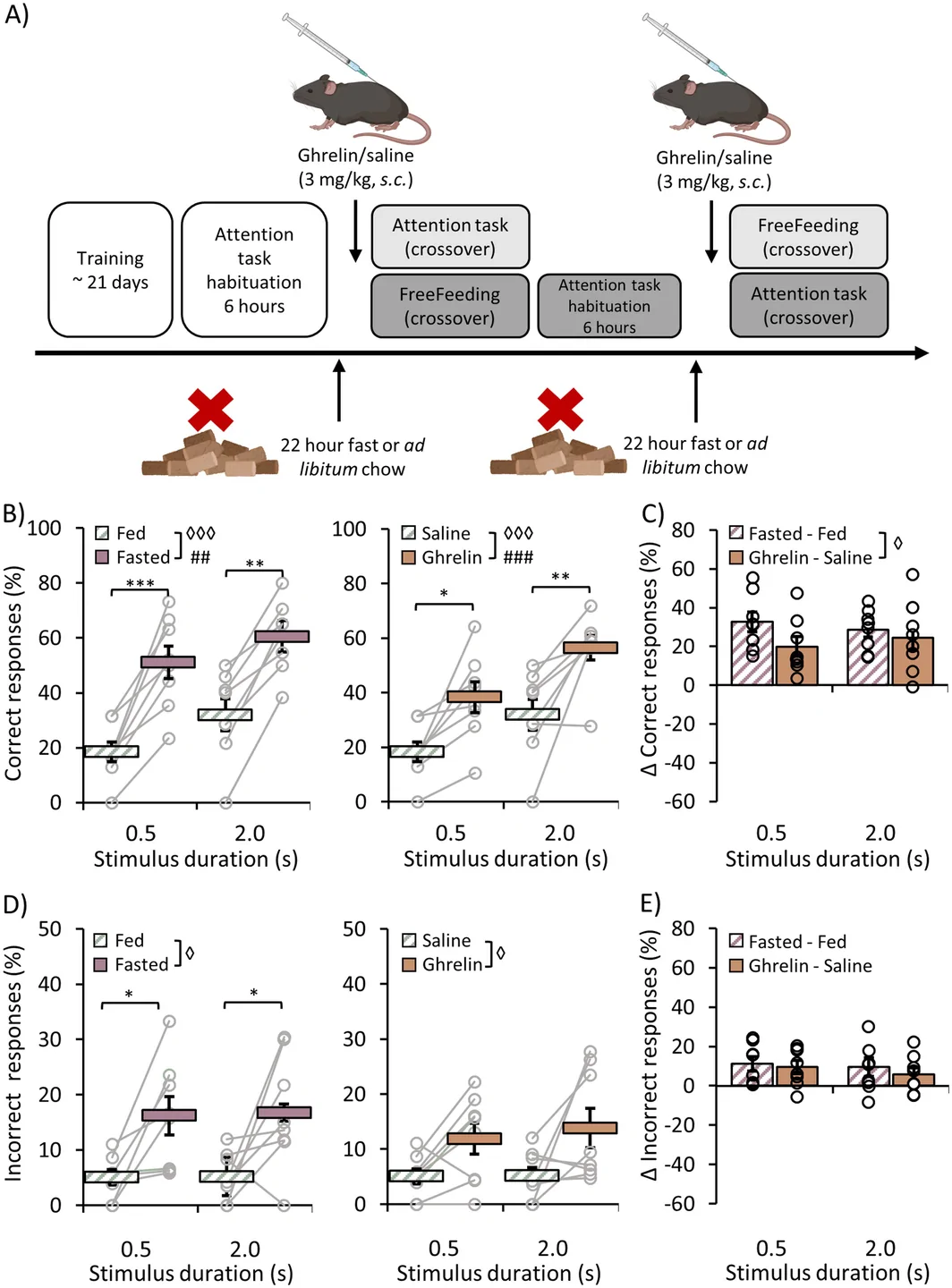
Fasting increases both correct and incorrect responses, whereas ghrelin selectively enhances correct responses in a conditioned food‐cue task. (A) Experimental design assessing food intake in the cue‐guided task and in the FreeFeeding session (in the absence of the illuminated predictive cue), following a 22 h fast or ghrelin administration. Mice were tested under fed + saline, fasted + saline, or fed + ghrelin conditions and exposed to either the cue‐guided task or FreeFeeding in the absence of an illuminated cue, in a crossover design. Mice assigned to the FreeFeeding condition were re‐habituated to the attention task before the cue‐guided task. (B) Correct responses to short (0.5 s) and long (2 s) light cue presentation expressed as a percentage of total trials during a 30 min attention session. (C) Change in correct responses relative to control conditions. (D) Incorrect responses expressed as a percentage of total trials. (E) Change in incorrect responses relative to control conditions. Mice served as their own controls (*n* = 8). Statistical analyses were performed using two‐way ANOVA. Symbols represent: ◊ *p* < 0.05; ◊◊ *p* ≤ 0.01; ◊◊◊ *p* ≤ 0.001 (general effect of treatment); *p < 0.05; **p ≤ 0.01; ***p ≤ 0.001 (fasted vs. fed or ghrelin vs. saline). Data are presented as mean ± SEM.

### 2.5 Data Analysis

Experimental data were retrieved from the FED3 devices and analyzed using custom Python graphical user interfaces (GUI; Python Software Foundation. Python Language Reference, version 3.12, http://www.python.org). FreeFeeding data were analyzed using FED3Viz, a customized GUI interface that was provided with the FED3 (Python Software Foundation. Python Language Reference, version 3.12, http://www.python.org). Data from the attention task were analyzed using a custom Python GUI developed in‐house. Attention task metrics included accuracy, omissions, premature responses and pellet retrieval latency. It also allowed for food intake analysis.

### 2.6 Experimental Design, Animal Welfare and Reporting

Reporting was prepared with reference to the ARRIVE guidelines. The individual mouse was the experimental unit for all analyses. The sample size was based on prior experience with within‐subject behavioral pharmacology studies using feeding‐related endpoints and was minimized in accordance with ethical approval while retaining a repeated‐measures crossover design. Treatment order and task order were counterbalanced across animals. Body weight, food intake and general health were monitored daily throughout training and testing. Supplemental chow was provided if mice completed fewer than 100 trials per session or fell below 90% of baseline body weight. Animals were removed from food‐restriction procedures if predefined welfare limits were reached. No pellet hoarding was observed during daily cage checks, and no adverse events or unexpected deaths occurred during the study. At the end of the experiment, animals were euthanized according to the approved protocol (Göteborgs djurförsöksetiska nämnd; permit number 2021‐3400, approved 6 December 2021). Adult male C57BL/6J mice were used to match prior ghrelin and operant‐feeding studies and to reduce biological variability in this initial validation of the task; the use of females will need to be addressed in future studies.

### 2.7 Statistics

Analyses were performed in IBM SPSS statistics 25 (IBM Corp., Armonk, NY, USA). Correct, incorrect and omission responses were analyzed using repeated measures two‐way ANOVA with treatment (saline‐fasted vs. saline‐fed or ghrelin‐fed vs. saline‐fed) and stimulus duration (0.5 and 2 s cue) as within‐subject variables. Significant main effects were followed up Tukey's HSD post hoc tests to evaluate pairwise differences. Paired *t*‐tests were used to analyze premature responses, pellet retrieval latency and differences in absolute food intake between the cue‐guided task and FreeFeeding. Plots were generated using Excel and show mean ± SEM. Statistical significance was set at *p* ≤ 0.05, and values 0.05 < *p* < 0.1 were considered statistical trends. Statistical annotations include the *p* value and its corresponding *F* or *t* ratio together with degrees of freedom.

## 3 Results

### 3.1 Establishment of a Novel Attention Task Using FED3 Feeding Devices

To evaluate attention‐linked behavior in response to a conditioned food‐cue, mice were trained to perform nose pokes in response to a light cue (an illuminated port) to obtain food pellets from FED3 devices placed in their home‐cages (Figure 1A,B). After 22 daily training sessions (Figure 1C), 13 mice met inclusion criteria (Figure 1A) and were tested in a 30‐min attention challenge session on each experimental day. During these sessions we recorded correct, incorrect, omission and premature responses to a short (SD 0.5 s) or long (SD 2 s) light cue presentation, as well as pellet retrieval latency, defined as the time taken to remove a dispensed pellet from the magazine; Figure 1B. Eight mice were included in the final data analysis; two mice were excluded after testing for outliers in correct‐, incorrect‐ and premature nose pokes, and three mice were excluded due to technical errors during the experiment.

### 3.2 Fasting and Ghrelin Enhance Attention‐Linked Responding to a Conditioned Food Cue

To assess the effects of fasting and ghrelin on attention task performance, mice were tested under three conditions: fed, overnight‐fasted and ghrelin‐treated. Fasted mice were food deprived for 22 h before testing, whereas fed and ghrelin‐treated mice had chow until 2 h before testing. Mice received saline or ghrelin 15 min before the 30‐min attention challenge task (Figure 2A).

Correct and incorrect responses were analyzed as measures of task performance, with correct responses also reflecting reinforced responding and food intake during the task. Repeated‐measures two‐way ANOVA revealed a significant main effect of fasting (*F*[1,7] = 142.174; *p* < 0.001), or ghrelin treatment (*F*[1,7] = 30.159; *p* = 0.001) on correct responses. Fasting increased correct responses to both short and long cue presentations compared with the fed condition (fasting vs. fed 0.5 s cue; *p* < 0.001; fasting vs. fed 2 s cue; *p* = 0.002, Figure 2B). Ghrelin treatment also increased correct responses compared with saline (ghrelin vs. saline 0.5 s cue; *p* = 0.037; ghrelin vs. saline 2 s cue; *p* = 0.007; Figure 2B). When changes from the respective control conditions were compared, the increase in correct responses was greater after fasting than after ghrelin treatment (*F*[1,7] = 7.017; *p* = 0.033; Figure 2C). Fasting also increased incorrect responses (*F*[1,7] = 11.159; *p* = 0.012) with increases evident for both cue durations (fasting vs. fed 0.5 s cue; *p* = 0.033; fasting vs. fed 2 s cue; *p* = 0.026; Figure 2D). Although ghrelin did not significantly increase incorrect responses, the changes in incorrect responses did not differ significantly between fasting and ghrelin treatment (*F*[1,7] = 0.790; *p* = 0.428; Figure 2E).

Omissions were interpreted as reflecting task engagement, with attentional and motivational components likely contributing because cues were presented passively. Repeated‐measures two‐way ANOVA showed significant effects of fasting (*F*[1,7] = 24.146; *p* = 0.002) and ghrelin treatment (*F*[1,7] = 28.143; *p* = 0.001) on omissions (Figure 3A). Both fasting and ghrelin reduced omissions compared with their respective control conditions (fasting vs. fed 0.5 and 2 s cue; *p* < 0.001; ghrelin vs. saline, 0.5 s: *p* < 0.001; 2 s: *p* = 0.001; Figure 3A). The change in omissions did not differ significantly between the fasting and ghrelin conditions, although there was a trend toward a greater reduction after fasting (*F*[1,7] = 5.489; *p* = 0.052; Figure 3B).

**FIGURE 3:**
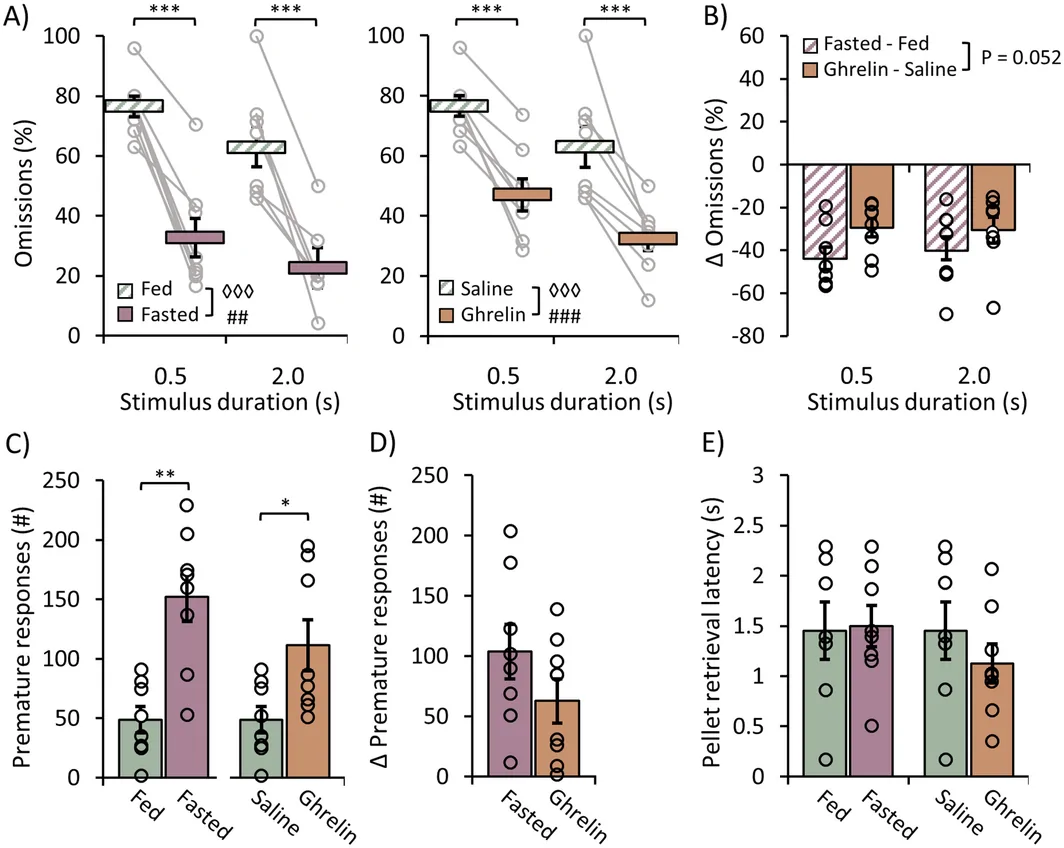
Fasting and ghrelin reduce omission responses and increase premature responses to a conditioned food cue during a 30‐min attention task session. (A) Omission responses to the short (0.5 s) and long (2 s) light cue presentation as a % of total trials in fasted and ghrelin‐injected mice. (B) Omission response change as a % of total trials of fasted and ghrelin‐injected mice compared to the control condition. (C) Number of premature responses and (D) premature response change of fasted and ghrelin‐injected mice compared to the control condition. (E) Average latency to retrieve pellet from the magazine. Mice served as their own controls (*n* = 8). Symbols represent: ◊ *p* < 0.05; ◊◊ *p* ≤ 0.01; ◊◊◊ *p* ≤ 0.001 (general effect of treatment); *p < 0.05; **p ≤ 0.01; ***p ≤ 0.001 (fasted vs. fed or ghrelin vs. saline). Data are presented as mean ± SEM.

Premature responses were considered alongside correct responses and omissions as part of the overall task‐engagement profile, while acknowledging possible contributions from motivation and impulse‐control processes. Both fasting and ghrelin significantly increased premature responses during the attention task (fasting vs. fed, *t*[7] = −4.591; *p* = 0.003; ghrelin vs. saline *t*[7] = −3.409; *p* = 0.011; Figure 3C). The magnitude of this increase was similar for fasting and ghrelin (*t*[7] = 1.521; *p* = 0.172; Figure 3D).

Pellet retrieval latency was measured to control for possible motor impairments or altered retrieval behavior. No significant differences were observed in either the fasting or ghrelin conditions (fasting vs. fed, *t*[6] = −0.795; *p* = 0.457; ghrelin vs. saline *t*[6] = 1.391; *p* = 0.214; Figure 3E).

### 3.3 Fasting and Ghrelin Increase Intake During the Cue‐Guided Task

To determine whether the observed effects of fasting and ghrelin on attention task performance were accompanied by changes in food intake, we measured food consumed during a 30‐min FreeFeeding session (Figure 4A) and during the 30‐min cue‐guided attention task (Figure 4B). When comparing the effect of fasting or ghrelin within each paradigm, both fasting and ghrelin increased absolute food intake during the cue‐guided task (Figure 4B). In contrast, during FreeFeeding, fasting, but not ghrelin, increased absolute food intake compared with the respective fed or saline control condition (FreeFeeding fasting vs. fed, *t*[7] = −11.39; *p* < 0.001; cue‐guided fasting vs. fed, *t*[7] = −8.186; *p* < 0.001; cue‐guided ghrelin vs. saline, *t*[7] = −4.647; *p* = 0.002; two‐sided paired sample *t*‐test).

**FIGURE 4:**
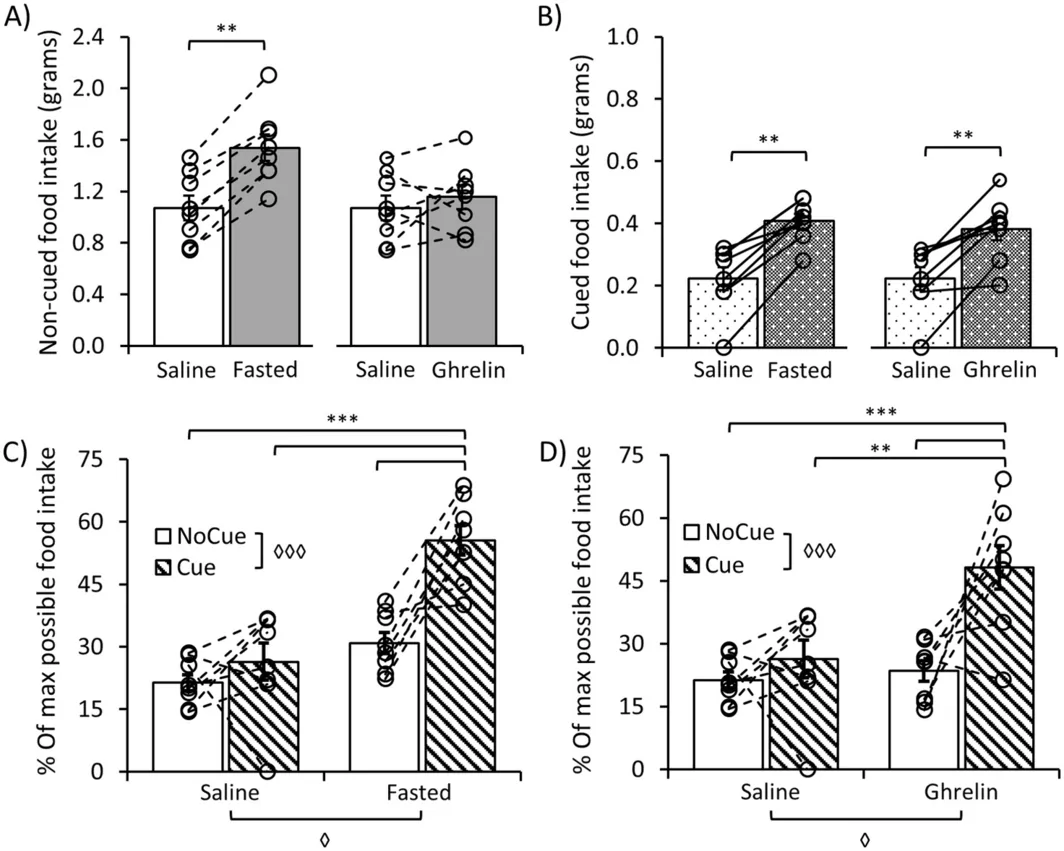
Ghrelin increases food intake during the cue‐guided task, and both fasting and ghrelin increase feeding rate in this context. (A, B) Average food intake after fasting or ghrelin injection during a 30‐min FreeFeeding session, in the absence of the illuminated predictive cue (A) and during the cue‐guided task. (C, D) Food intake normalized to the maximum possible food intake (opportunity) for each individual mouse during the FreeFeeding session or the cue‐guided task following fasting (C) or ghrelin injection (D). Mice served as their own controls (*n* = 8). Symbols represent: ◊ *p* < 0.05; ◊◊ *p* ≤ 0.01; ◊◊◊ *p* ≤ 0.001 (general effect of task context); *p < 0.05; **p ≤ 0.01; ***p ≤ 0.001. Data are presented as mean ± SEM.

To compare intake between the cue‐guided task and FreeFeeding, we accounted for the different number of opportunities to obtain pellets, since the cue‐guided task included an ITI. For each mouse, the maximum obtainable intake during the cue‐guided task was estimated from its average feeding rate during the saline + fed FreeFeeding session and the total number of trials completed during the saline cue‐guided session. Food intake was then expressed as a percentage of this maximum obtainable intake. Repeated two‐way ANOVA showed significant effects of task context (Figure 4C: fasting vs. saline: *F*[1,7] = 142.211; *p* < 0.001; Figure 4D: ghrelin vs. saline: *F*[1,7] = 28.181; *p* = 0.001), and treatment (Figure 4C: fasting vs. saline: *F*[1,7] = 9.395; *p* = 0.018; Figure 4D: ghrelin vs. saline: *F*[1,7] = 7.334; *p* = 0.030). Both fasting and ghrelin significantly increased the proportion of maximum obtainable intake during the cue‐guided task, compared with their respective control conditions (Fasting/cue‐guided vs. saline/cue‐guided *p* < 0.001; Fasting/cue‐guided vs. fasting/FreeFeeding *p* < 0.001; Fasting/cue‐guided vs. saline/FreeFeeding: *p* < 0.001; Ghrelin/cue‐guided vs. saline/cue‐guided *p* = 0.002; Ghrelin/cue‐guided vs. Ghrelin/FreeFeeding *p* < 0.001; Ghrelin/cue‐guided vs. saline/FreeFeeding: *p* < 0.001). Thus, ghrelin did not increase intake during FreeFeeding, in the absence of the illuminated predictive cue, whereas fasting increased intake in this condition. This suggests that, under the present experimental conditions, ghrelin's effect on food intake was preferentially expressed during the cue‐guided task, while fasting increased intake across both task contexts.

## 4 Discussion

The present study describes a novel home‐cage FED3‐based paradigm for assessing behavioral responses to a conditioned food cue and uses this approach to examine the effects of fasting and ghrelin. Both acute ghrelin and fasting increased correct responding, reduced omissions and increased premature responses during cue‐guided testing, consistent with enhanced engagement with the conditioned food cue, although attentional, motivational and impulse‐control processes are all likely to contribute to this behavioral profile. Despite these similarities, fasting and ghrelin differed in their effects on food intake. Fasting increased intake across both cue‐guided testing and FreeFeeding, whereas ghrelin increased intake only during the cue‐guided task under the present experimental conditions. Together, these findings suggest that fasting and acute ghrelin produce overlapping effects on cue‐guided behavior but differ in how feeding is expressed across task contexts.

A central observation was that acute ghrelin did not fully reproduce the behavioral effects of fasting. Although fasting is accompanied by elevations in circulating ghrelin (Muller et al. 2002; Tschöp et al. 2000), it also engages a wide range of additional endocrine, metabolic and neural adaptations. This distinction is important because it suggests that the effects observed after fasting cannot be attributed to ghrelin alone. While both manipulations enhanced engagement with the conditioned food cue, fasting additionally increased food intake during FreeFeeding, consistent with a broader physiological state that promotes consumption across contexts. In contrast, the effects of ghrelin were expressed preferentially during the cue‐guided task.

The present findings add to a growing literature linking ghrelin signaling to conditioned and learned aspects of feeding behavior. Previous studies have shown that ventral hippocampal ghrelin signaling promotes feeding elicited by conditioned food‐predictive stimuli (Kanoski et al. 2013), while disruption of ghrelin receptor signaling reduces cue‐potentiated feeding (Walker et al. 2012). Human neuroimaging studies similarly demonstrate associations between circulating ghrelin concentrations and neural responses to food cues (Kroemer et al. 2013; Malik et al. 2008; Wever et al. 2021). Our findings extend these observations by providing a behavioral assay that resolves multiple components of cue‐guided responding, including correct responses, omissions, premature responses and food intake within the same task framework.

A major objective of this study was to assess attention to a conditioned food‐predictive cue. The task was designed as an attention challenge, requiring mice to detect and respond to brief, unpredictable cue presentations. Increased correct responding together with reduced omissions therefore provides evidence that fasting and ghrelin enhanced cue‐directed attentional engagement. At the same time, attention task performance is necessarily shaped by interacting processes, including motivation, arousal and inhibitory control (Asinof and Paine 2014; Aston‐Jones and Cohen 2005; Robbins 2002). This is especially relevant here because the attended stimulus predicted food availability. Thus, we interpret the behavioral effects of fasting and ghrelin as enhanced attention‐linked responding to the food cue, with the increase in premature responses indicating that motivation and impulse‐control processes also shaped task performance.

The comparison between cue‐guided testing and FreeFeeding offers additional insight into the context dependence of ghrelin‐induced feeding. Notably, ghrelin increased food intake during the cue‐guided task but not during FreeFeeding. This finding is consistent with the idea that ghrelin amplifies behavioral responses when food availability is organized around a predictive stimulus. However, this interpretation requires caution. First, baseline intake during FreeFeeding was high, which may have limited sensitivity to detect a further ghrelin‐induced increase in this condition. We therefore also examined intake relative to the maximal obtainable intake in each task context, to account for differences in feeding opportunity between FreeFeeding and the cue‐guided task. Ghrelin increased this normalized measure during the cue‐guided task but not during FreeFeeding. This suggests that the absence of a ghrelin effect on absolute FreeFeeding intake may partly reflect high baseline intake while still supporting a preferential effect of ghrelin on feeding expressed during cue‐guided testing. Second, the cue‐guided task and FreeFeeding condition differed not only in the presence of the illuminated predictive cue, but also in the requirement for an operant response and in the overall task structure. Third, FreeFeeding should not be considered entirely cue‐free because dispensed food pellets provide sensory feedback once delivered. However, this feedback is qualitatively different from the discrete illuminated cue in the attention task, which predicts food availability and controls responding. Thus, although FreeFeeding contains food‐related sensory information, it lacks the strong predictive cue that structures behavior in the cue‐guided task.

Both fasting and ghrelin also increased premature responding. In traditional operant paradigms, premature responses are often interpreted as impaired inhibitory control or increased impulsivity (Asinof and Paine 2014; Robbins 2002). However, because animals did not initiate trials in the present task, premature responses may also reflect perseverative responding or more general increases in behavioral activation. The parallel increase in premature responses, reduction in omissions and increase in correct responding suggests a broader increase in task engagement rather than a selective change in impulsivity alone. Consistent with this interpretation, ghrelin has previously been reported to increase impulsive behavior in rodents (Anderberg et al. 2016), while also enhancing food motivation and reward‐related behaviors (Perello et al. 2010; Skibicka et al. 2011, 2012).

The FED3 platform enabled assessment of food‐cue responsiveness in the home cage with minimal experimenter intervention while simultaneously capturing both operant behavior and food intake. This makes the approach particularly suitable for studies involving genetically modified mice or long‐term manipulations. Several limitations should also be acknowledged. The study was conducted in male mice only, response latency to the cue was not measured, and the design does not allow complete separation of attentional, motivational and arousal‐related influences on behavior. In addition, the cue‐guided task and FreeFeeding condition differed in multiple respects beyond cue presentation. Future studies that independently manipulate predictive cues and response requirements, together with direct measurements of response latency and neural activity, may help define the mechanisms through which ghrelin influences cue‐guided feeding behavior.

## 5 Conclusion

In conclusion, we developed a home‐cage operant paradigm for assessing attention‐linked behavioral responses to a conditioned food cue in mice. Fasting and ghrelin produced overlapping effects on task performance, including increased correct responding, reduced omissions and increased premature responding, consistent with enhanced cue‐oriented task engagement. Their effects on intake, however, differed: fasting increased intake across both cue‐guided testing and FreeFeeding, whereas ghrelin preferentially increased intake during cue‐guided testing under the present experimental conditions. This divergence suggests that fasting produces a broader hunger state that promotes consumption across contexts, while acute ghrelin may more selectively amplify feeding when behavior is organized around food‐predictive cues. These findings build on previous work linking ghrelin signaling to food‐cue responsiveness and extend it by resolving multiple behavioral components within a single home‐cage framework. They support the idea that internal hunger‐related signals interact with external food‐predictive cues to shape behavioral engagement and consumption, while highlighting the need for future studies to define the neural mechanisms through which ghrelin modulates cue‐guided feeding behavior.

## Funding

Funding of this study was provided by Swedish Research Council for Medicine and Health (2022‐00713, 2024‐02295 to SLD), Hjärnfonden (FO2024‐0072, FO2025‐0051 to SLD), Novonordisk Fonden (NNF0078215 and NNF0104484 to SLD), ALF‐GBG (ALFGBG‐1005655 to SLD), Dutch Research Council NWO (ALWOP.137, OCENW.M.22.111 to RAHA and Gravitation grant 024.004.012 to RAHA). We also acknowledge the Nutrition Network of the European College for Neuropsychopharmacology for facilitating this collaboration through networking support. The Swedish Research Council for Medicine and Health, Hjärnfonden, Novonordisk Fonden, ALF‐GBG and Dutch research Council NWO had no further role in study design; in the collection, analysis and interpretation of data; in the writing of the report; and in the decision to submit the paper for publication.

## Conflicts of Interest

The authors declare no conflicts of interest.

## Data Availability

The data that support the findings of this study are available on request from the corresponding author. The data are not publicly available due to privacy or ethical restrictions.
